# Supplementary material for: Beta3-Tubulin Is Critical for Microtubule Dynamics, Cell Cycle Regulation, and Spontaneous Release of Microvesicles in Human Malignant Melanoma Cells (A375)
Source: Int J Mol Sci. 2020 Feb 28;21(5):1656. doi: 10.3390/ijms21051656 (PMC7084453; doi:10.3390/ijms21051656)
Supplement: Supplementary file 1 [file ijms-21-01656-s001.pdf]

Supplementary data

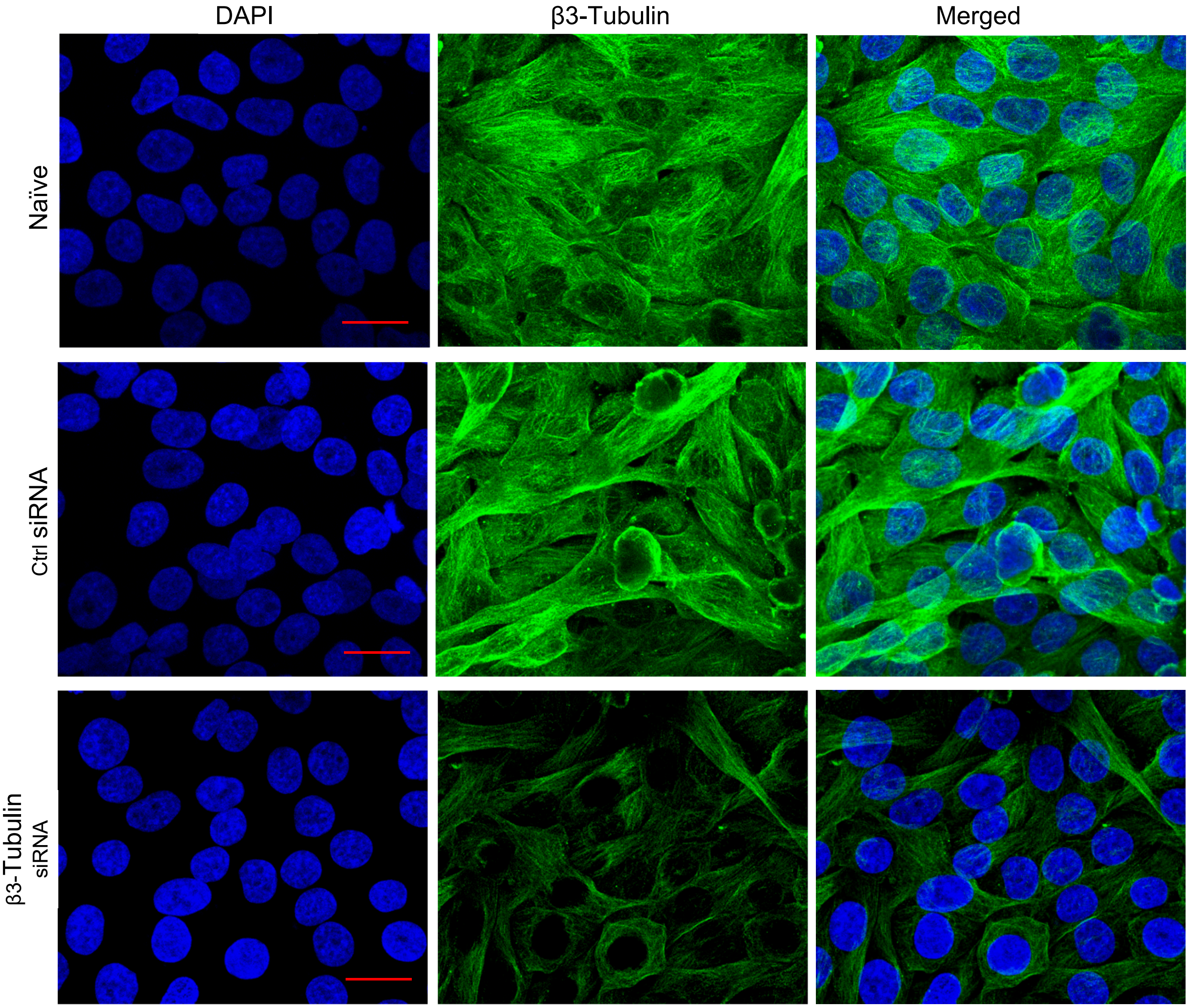

Supplemental figure S1. siRNA knockdown of β3-tubulin in A375 melanoma cells. Naïve, control (ctrl)-, and β3-tubulin siRNA-treated cells were immunostained with antibodies specific for β3-tubulin (green), and DAPI (blue) was used as nuclear DNA counterstain. Original magnification × 630 (bar, 20 μm).

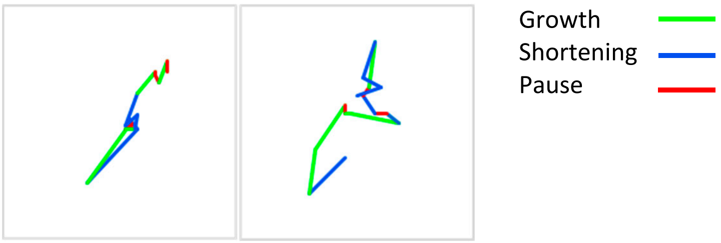

Supplemental figure S2. Two representative traces of the Microtubules (MT) +ends displacement obtained by computer-assisted hand clicking using ImageJ software (Manual tracking plugin, https://imagej.nih.gov/ij/plugins/track/track.html).

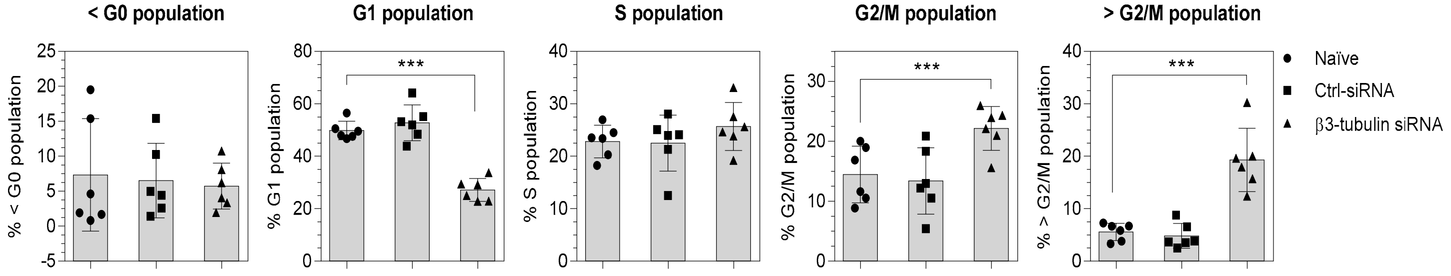

Supplemental figure S3. Cell cycle analysis. β3-tubulin knockdown redistributed cell cycle growth phases and induced G2/M cell cycle arrest. A375 melanoma cells were stained with Propidium Iodide (PI) and Cell cycle analysis was performed using a flow cytometer (Guava® easyCyte; MilliporeSigma). Data analysis was carried out using FlowJo v10.6.1 software. Statistical significance was determined between different groups using ANOVA with Tukey's correction for multiple comparisons. $N = 6$, ***, $P < 0.001$. versus naïve cells.

Supplemental table S1: MT dynamic parameters in the human skin malignant melanoma calls (A375). Data demonstrate the difference in MT+ends growth, shortening, pause frequency and dynamicity among naïve, control, β3-tubulin siRNA transfected cells. Asterisks indicate *, $P < 0.05$, ***, $P < 0.001$ Student t-test versus naïve values.

Naïve					
**Microtubules (MT)**	**Growth (µm/min)**	**Shortening (µm/min)**	**Pause frequency (min^{-1})**	**Total MT+ends distance +, growth; -, shortening (µm)**	**Dynamicity (µm/min)**
MT1	35.67	29.36	32	-1.57	65.03
MT2	57.82	27.99	36	-7.45	85.82
MT3	18.55	32.22	32	3.41	50.78
MT4	22.54	49.05	28	6.62	71.59
MT5	17.97	39.28	36	5.32	57.25
MT6	34.78	30.58	32	-1.04	65.36
MT7	38.66	45.82	36	4.79	84.48
MT8	31.15	20.46	40	-2.67	51.62
MT9	21.22	47.69	28	6.61	68.91
MT10	44.41	28.46	36	-3.98	72.87
**Mean**	**32.277**	**35.091**	**33.6**	**1.004**	**67.371**
**Control siRNA**					
**Microtubules (MT)**	**Growth (µm/min)**	**Shortening (µm/min)**	**Pause frequency (min^{-1})**	**Total MT+ends distance +, growth; -, shortening (µm)**	**Dynamicity (µm/min)**
MT1	35.38	20.44	44	3.73	55.82
MT2	41.78	32.05	24	2.43	73.84
MT3	28.53	42.62	40	-3.52	71.16
MT4	23.50	29.19	36	-1.42	52.71
MT5	25.56	20.91	32	1.16	46.47
MT6	51.36	22.08	32	7.32	73.45
MT7	38.46	25.15	32	3.32	63.62
MT8	40.39	33.11	32	1.82	73.51
MT9	41.64	43.33	32	-0.42	84.98
MT10	38.35	51.76	40	-3.35	90.12
**Mean**	**36.495**	**32.064**	**34.4**	**1.107**	**68.568**
**β3-tubulin siRNA**					
**Microtubules (MT)**	**Growth (µm/min)**	**Shortening (µm/min)**	**Pause frequency (min^{-1})**	**Total MT+ends distance +, growth; -, shortening (µm)**	**Dynamicity (µm/min)**
MT1	12.38	18.19	40	-1.45	30.58
MT2	18.27	19.35	32	-0.26	37.62
MT3	16.06	19.19	44	-0.78	35.25
MT4	17.26	14.31	44	0.74	31.56
MT5	17.77	17.43	40	0.08	35.21
MT6	19.44	20.23	36	-0.19	39.67
MT7	18.04	14.41	44	0.91	32.46
MT8	21.31	19.97	36	0.33	41.28
MT9	23.27	9.93	44	3.33	33.21
MT10	30.74	12.64	40	3.52	43.38
**Mean**	**19.454*** **	**16.565*** **	**40***	**0.623*** **	**36.022*** **
